# 

**Published:** 1893-03

**Authors:** 


### THE BRISTOL

### Medico-Chirurgical Journal.

A JOURNAL OF THE MEDICAL SCIENCES FOR THE WEST OF ENGLAND AND SOUTH WALES

*PUBLISHED UNDER THE AUSPICES OF*

*THE BRISTOL MEDICO-CHIRURGICAL SOCIETY.*

EDITOR:

R. SHINGLETON SMITH, M.D., B.Sc.

WITH WHOM ARE ASSOCIATED

BARCLAY J. BARON, M.B., C.M.

J. MICHELL CLARKE, M.A., M.D.

F. R. CROSS, M.B.

W. H. HARSANT.

ASSISTANT-EDITOR:

L. M. GRIFFITHS.

"Scire est nescire, nisi id me
Scire alius sciret."

*VOL. XI.*

BRISTOL: J. W. ARROWSMITH;
LONDON: J. & A. CHURCHILL, 11 NEW BURLINGTON STREET.
1893.

L.H.

R31
B8

J.W. Arrowsmith

Publisher

Bristol.

### CONTENTS OF VOLUME XI.

*Page*

Acute Inflammation around the Upper Cervical Vertebræ. A Subjective Clinical Study. By JOHN KENT SPENDER, M.D. Lond. 1

Pleural Effusions: Their Diagnosis and Treatment. By ALFRED SHEEN, M.D. St. And. ... ... ... ... ... ... ... ... ... 14

A Case of Extra-Uterine Gestation. By A. E. AUST LAWRENCE, M.D. Aberd., and CHARLES A. MORTON, F.R.C.S. Eng. ... 29

Asthenopia and Ocular Headache. By F. R. CROSS, F.R.C.S. Eng., M.B. Lond. ... ... ... ... ... ... ... ... ... ... 73

A Case of Thrombosis of the Femoral Artery Leading to Gangrene. By ARTHUR E. BARKER, F.R.C.S. Eng. ... ... ... ... ... 84

Two Cases of Branchial Fistulæ. By GEORGE PARKER, M.D., M.A. Cantab. ... ... ... ... ... ... ... ... ... ... ... 89

One View of Heredity. By AUGUSTIN PRICHARD, F.R.C.S. Eng., M.D. Berlin ... ... ... ... ... ... ... ... ... ... 91

The Alleged Increase of Insanity. By C S. W. COBBOLD, M.D. Würz., F.R.C.P. Ed. ... ... ... ... ... ... ... ... ... ... 145

The Combination of Syphilis and Tuberculosis, especially in regard to Laryngeal Affections. By P. WATSON WILLIAMS, M.D. Lond. ... ... ... ... ... ... ... ... ... ... ... ... 155

Lesion of the Cauda Equina: Operation—Relief of Symptoms. By J. E. SHAW, M.B. Edin., and J. PAUL BUSH, M.R.C.S. Eng. ... ... ... ... ... ... ... ... ... ... ... ... ... 161

Rötheln: a Summary. By JOHN MEREDITH, M.D. Ed. ... ... 171

Modern Medical Journalism. By J. GREIG SMITH, M.A., M.B., C.M. Aberd., F.R.S.E. ... ... ... ... ... ... ... ... ... 217

Compression of the Umbilical Cord during Forceps Delivery. By Joseph Griffiths Swayne, M.D. Lond. ... ... ... ... ... 229

A Case of Double Empyema. By William Johnstone Fyffe, B.A., M.D. Dub. ... ... ... ... ... ... ... ... ... ... 233

Three Cases of Umbilical Hemorrhage occurring in the Same Family. By James Taylor, M.R.C.S. Eng. ... ... ... ... 237

Health-Resorts in the West of England and South Wales.
Torquay. By Paul Q. Karkeek, M.R.C.S. Eng., L.S.A. ... 93
Cheltenham. By J. H. Garrett, M.D., B.S. Dur., D.P.H. Camb. ... ... ... ... ... ... ... ... ... ... ... .. 241

Progress of the Medical Sciences ... ... 32, 100, 175, 253

Reviews of Books ... ... ... ... ... ... ... ... 46, 113, 190, 270

Notes on Preparations for the Sick ... ... 60, 132, 210, 277

Meetings of Societies ... ... ... ... ... ... ... ... 62, 135, 281

The Library of the Bristol Medico-Chirurgical Society ... ... ... ... ... ... ... ... ... ... 64, 137, 212, 282

The Hodgkins Fund Prizes ... ... ... ... ... ... ... ... 141

The Bristol Eye Hospital ... ... ... ... ... ... ... ... ... ... 142

University College, Bristol: Faculty of Medicine ... ... 266

Scraps ... ... ... ... ... .. ... ... ... ... ... 71, 143, 215, 287

### The Library of the Bristol Medico-Chirurgical Society.

*The following donations have been received since the publication of the list in December :*

*February 28th*, 1893.

| | |
|---|---|
| John Beddoe, M.D. (1) ... ... ... ... ... ... | 2 volumes. |
| British Medical Association (2) ... ... ... ... | 163 „ |
| C. F. Pickering (3) ... ... ... ... ... ... ... | 4 „ |
| W. E. Pountney, M.D. (4) ... ... ... ... ... | 4 „ |
| Bertram M. H. Rogers, M.D. (5) ... ... ... ... | 1 volume. |
| The Royal Medical and Chirurgical Society (6) ... | 36 volumes. |
| The Surgeon-General, United States Army (7) ... | 1 volume. |

Unbound periodicals have been received from the British Medical Association, Dr. Michell Clarke, and Mr. F. R. Cross.

Framed pictures have been presented by Mr. R. W. Coe and Dr. Barrett Roué.

### SEVENTH LIST OF BOOKS.

The figures in brackets refer to the figures after the names of the donors, and show by whom the volumes were presented. The books to which no such figures are attached have either been bought from the Library Fund or received through the *Journal*.

| | | | |
|---|---|---|---|
| **Althaus, J.** ... ... | *Influenza* ... ... ... ... ... ... ... ... | 2nd Ed. | 1892 |
| **Armstrong, J.** ... | *The Elements of Pathology* ... ... ... ... ... ... | (2) | 1838 |
| **Ballantyne, J. W.**... | *The Diseases of the Fœtus* ... ... ... ... ... ... | | [1892] |
| **Ballard, E.** ... ... | *Diseases of the Abdomen* ... ... ... ... ... ... | (2) | 1852 |
| **Ballard and A. B.** | **Garrod, E.** *Elements of Materia Medica and Therapeutics* ... ... .. ... ... ... ... | (2) | 1845 |
| **Bartlett, H. C.** ... | *The Digestion and Assimilation of Fat in the Human Body* ... ... ... ... ... .. ... . . ... | (2) | 1877 |
| **Bastian, H. C.** ... | *Hysterical or Functional Paralysis* ... ... ... ... | | 1893 |

Bayle and H. Hollard, A. L. J. *Manual of General Anatomy.* (Tr. by H. Storer) ... ... ... ... ... ... ... ... (2) 1829

Begbie, J. W. ... *Works.* (Ed. by D. Duckworth) ... ... ... ... 1882

Belloste, A. ... ... *The Hospital Surgeon* ... .. ... ... (4) 4th Ed. 1732

Bichat, X. ... ... *Physiological Researches on Life and Death.* (Tr. by F. Gold) ... ... ... ... ... ... ... ... (2) [N.D.]

Bigg, R. H. ... ... *Spinal Curvature* ... ... ... ... ... ... ... (2) 1882

" ... ... *Artificial Limbs* ... ... ... ... ... .. ... (2) 1885

Billroth, T. ... ... *Clinical Surgery.* (Tr. and Ed. by C. T. Dent) ... 1881

Blanc, H. ... ... *Advice to Intending Visitors to Cannes* ... ... ... 1893

Blumenbach, J. F.... *A Manual of Comparative Anatomy.* (Tr. by W. Lawrence) ... ... ... ... ... (2) 2nd Ed. 1827

Bostock, J. ... ... *An Elementary System of Physiology*... (2) [3rd Ed. 1836]

Bright, R. ... ... *Clinical Memoirs on Abdominal Tumours.* (Ed. by G. H. Barlow) ... ... ... ... ... ... (2) 1860

Brodie, B. C. ... ... *On Diseases of the Joints* ... ... ... (2) 2nd Ed. 1822

" ... ... *On the Urinary Organs* ... ... ... (2) 3rd Ed. 1842

Burggraeve, A. ... *The New Handbook of Dosimetric Therapeutics.* (Tr. and Ed. by H. A. Allbutt) ... ... ... (4) 1882

Bury, J. Ross and J. S. *A Treatise on Peripheral Neuritis* .. ... ... ... 1893

Carpenter, W. B. ... *Principles of Human Physiology* ... ... ... (2) 1842

Casper, J. L. ... ... *Forensic Medicine.* (Tr. by G. W. Balfour.) Vols. I., II. ... ... ... ... ... ... (2) 1861–62

*Catalogue (Index) of Library of Surgeon-General's Office, U.S. Army.* Vol. XIII. ... ... ... ... ... ... ... ... (7) 1892

Celsus, A. C. ... ... *De Re Medica.* The first four books (with Tr. by J. Steggall.) ... ... ... ... ... ... (3) 2nd Ed. 1853

Cerna, D. ... ... *Notes on the Newer Remedies* ... ... ... ... ... 1893

Chapman, H. C. ... *Medical Jurisprudence and Toxicology* ... ... ... 1892

Charcot, J. M. .. *Diseases of the Nervous System.* (Tr. by G. Sigerson) 1877

" ... " " 2nd Series ... ... 1881

" ... *Lectures on Senile Diseases.* (Tr. by W. S. Tuke)... 1881

Cheyne, G. ... ... *The Natural Method of Cureing the Diseases of the Body, and the Disorders of the Mind depending on the Body* ... ... ... ... ... ... ... (2) 2nd Ed. 1742

Cheyne, J. ... ... *Cases of Apoplexy and Lethargy*... ... ... ... (2) 1812

Christison, R. ... *A Treatise on Poisons* ... ... ... ... (2) 3rd Ed. 1836

Churchill, F.... ... *Diseases of Pregnancy and Childbed* ... ... ... (2) 1840

" ... ... *Researches on Operative Midwifery* ... ... ... (2) 1841

Clarke, E. G. ... ... *Medicinæ Praxeos Compendium.* Editio tertia... (2) 1805

Conquest, J. T. ... *Outlines of Midwifery* ... ... ... ... (2) 6th Ed. 1837

Constant, T. E. ... *How to Give Gas* ... ... ... ... ... ... ... ...[1892]

Cooper, Sir A. ... *Lectures on the Principles and Practice of Surgery* (2). 8th Ed. 1835

Cooper, B. B. ... ... *Surgical Essays* ... ... ... ... ... ... ... (2) 1833

" ... ... *Principles and Practice of Surgery* ... ... ... (2) 1851

Cooper, S. ... ... *Dictionary of Practical Surgery* ... ... (2) 5th Ed. 1825

Coster, J. ... ... *Manual of Operative Surgery.* (Tr. by G. Fife) (2) 3rd Ed. 1831

**Cozzolino, V.** ... ... *The Hygiene of the Ear.* 5th Ed. (Tr. by J. Erskine) 1892
**Crosse, W. H.** ... *Notes on Malarial Fevers* ... ... ... ... ... ... ... 1892
**Cullen, W.** ... ... *Synopsis Nosologiæ methodicæ* (Latin and English) (2) 1813-23
**Curling, T. B.** ... *Diseases of the Rectum*... ... ... ... ... ... ... (2) 1851
**Cutler, T.** ... ... *The Surgeon's Practical Guide* ... ... (2) 2nd Ed. 1836
**Czermak, J. N.** ... *Practical Uses of the Laryngoscope.* (Tr. by G. D. Gibb) ... ... ... ... ... ... ... ... (2) 1861

**Davis, N. S., jun.** ... *Diseases of the Lungs, Heart, and Kidneys* ... ... 1892
**Dewey, M.** ... ... *Decimal Classification* ... ... ... ... ... ... 4th Ed. 1891
**Diday, P.** ... ... *Infantile Syphilis.* (Tr. by G. Whitley) ... (2) 1859
*Diphtheria, Memoirs on.* (Tr. by R. H. Semple)... ... ... ... ... (2) 1859
**Dusch, T. v.** ... ... *Thrombosis of the Cerebral Sinuses.* (Tr. by G. Whitley) ... ... ... ... ... ... ... ... ... (2) 1861

**Ellis, A.** ... ... ... *Clinical Surgery* ... ... ... ... ... ... ... ... (2) 1846
**Ellis, G. V.** ... ... *Demonstrations of Anatomy* ... ... ... ... (2) 1840
**Ellis, Sir W. C.** ... *Insanity* .. ... ... ... ... ... ... ... (2) 1838
*Entomology, Letters on*... ... ... ... ... ... ... ... ... ... ... ... (4) 1825
**Esmarch, F.** ... ... *The Uses of Cold in Surgical Practice.* (Tr. by E. Montgomery) ... ... ... ... ... ... ... (2) 1861
**Ewart, W.** ... ... *Symptoms and Physical Signs* ... ... ... ... ... 1892

**Fergusson, W.** ... *Practical Surgery* ... ... ... ... ... (2) 3rd Ed. 1852
**Feuchtersleben, Baron** *Medical Psychology.* (Tr. by H. E. Lloyd and Ed. by B. G. Babington) ... ... ... ... (2) 1847
**Forsbrook, W. H. R.** *Osteo-Arthritis* ... ... ... ... ... ... ... ... ... 1893
**Fownes, G.** ... ... *Elementary Chemistry* ... ... ... ... (2) 5th Ed. 1854
**Frerichs, F. T.** ... *Clinical Treatise on Diseases of the Liver.* (Tr. by C. Murchison.) 2 vols. ... ... ... (2) 1860-61
**Fyfe, A.** ... ... ... *A Compendium of Anatomy.* 4 vols. (2) 9th Ed. 1826

**Garrod and E. Ballard, A. B.** *Elements of Materia Medica and Therapeutics.* (2) 1845
**Gooch, R.** ... ... *On Diseases of Women and Children.* (Prefatory Essay by R. Ferguson)... ... ... ... ... (2) 1859
**Gowers, W. R.** ... *Syphilis and the Nervous System*... ... ... ... ... 1892
**Granville, A. B.** ... *On Abortion* ... ... ... ... ... ... ... ... ... (2) 1834
**Graves, R. J.** ... ... *A System of Clinical Medicine* ... ... ... ... (2) 1843
**Gregory, G.** ... ... *Elements of the Theory and Practice of Medicine* (2) 4th Ed. 1835
**Gregory, J.** ... ... *Conspectus Medicinæ Theoreticæ.* Editio Sexta. (2) 1818
" ... ... " " (Tr. by J. Steggall) (2) 4th Ed. 1837, (3) 5th Ed. 1855
**Griffith, W.** ... ... *A Treatise on Hydrocephalus* ... ... ... ... (2) 1835
**Guthrie, G. J.** ... *On the Urinary and Sexual Organs.* (2) 3rd Ed. 1843
" ... ... *Wounds and Injuries of the Abdomen* ... ... ... (2) 1847

**Hall, J. C.** ... ... *The Animal Kingdom* ... ... ... ... ... ... (2) 1841
**Hall, M.** ... ... ... *On the Constitutional Diseases of Females* (2) 2nd Ed. [1830]

Hamilton, A.... ... *Outlines of the Theory and Practice of Midwifery.* (2) 4th Ed. 1796

Harrison, R. ... ... *The Surgical Anatomy of the Arteries.* 2 vols. (2) 3rd Ed. 1833

" ... ... *The Dublin Dissector* ... ... ... ... (2) 4th Ed. 1836

Hasse, C. E. ... ... *Pathological Anatomy.* (Tr. and Ed. by W. E. Swaine) ... ... ... ... ... ... ... ... (2) 1846

Hecker, J. F. C. ... *The Epidemics of the Middle Ages.* (Tr. by B. G. Babington) ... ... ... ... ... ... ... (2) 1846

Hirsch, A. ... ... *Handbook of Geographical and Historical Pathology.* (Tr. by C. Creighton.) 3 vols. ... 2nd Ed. 1883-86

Hobart, Rev. W. K. *The Medical Language of St. Luke* ... ... ... ... 1882

Holden, L. ... ... *Manual of the Dissection of the Human Body.* (Ed. by J. Langton.) ... ... ... ... (2) 3rd Ed. 1868

Holland, H. ... ... *Medical Notes and Reflections* ... ... (2) 2nd Ed. 1840

Hollard, A. L. J. Bayle and H. *Manual of General Anatomy.* (Tr. by H. Storer) ... ... ... ... ... ... ... (2) 1829

Holmes, J. P.... ... *On Diseases Incident to Females* ... ... ... ... (2) 1831

Home, E. ... ... *Practical Observations on the Treatment of Strictures in the Urethra* ... ... ... ... ... ... ... (2) 1795

Hooper, R. ... ... *The Anatomist's Vade-mecum* ... ... (2) 9th Ed. 1820

Hunt, T. ... ... *On the Skin* ... ... ... ... ... ... ... (2) 6th Ed. 1862

Hunter, J. ... ... *A Treatise on the Blood, Inflammation, and Gunshot Wounds.* 2 vols. ... ... ... ... ... ... (2) 1812

Johnson, J. ... ... *The Morbid Sensibility of the Stomach and Bowels* (2) 4th Ed. 1827

Johnston, J. F. W. *Agricultural Chemistry and Geology* ... (2) 7th Ed. 1856

Johnstone, J.... ... *Materia Medica* ... ... ... ... ... ... ... (2) 1835

Keill, J. ... ... ... *The Anatomy of the Human Body Abridg'd* (2) 10th Ed. 1738

Kirkes, W. S. (assisted by J. Paget.) *Handbook of Physiology* ... (2) 1848

Kramer, W. ... ... *On Diseases of the Ear.* (Tr. by H. Power) ... (2) 1863

Küchenmeister, F.... *Manual of Parasites.* Vol. II. (Tr. by E. Lankester) ... ... ... ... ... ... ... (3) 1857

Kussmaul and A. Tenner, A. *On Epileptiform Convulsions from Hæmorrhage* (Tr. by E. Bronner) ... ... (2) 1859

Laennec, R. T. H.... *A Treatise on Mediate Auscultation, and on Diseases of the Lungs and Heart.* (Ed. by T. Herbert) (3) 1846

Landau, L. ... ... *Moveable Kidney in Women.* (Tr. and Ed. by F. H. Champnays) ... ... ... ... ... ... 1884

Lane, J. H. ... ... *Compendium of Materia Medica*... ... (2) 2nd Ed. 1851

Langrish, B. ... ... *The Modern Theory and Practice of Physic* (2) 2nd Ed. 1738

Lea, A. S. ... ... *The Chemical Basis of the Animal Body* ... ... ... 1892

Liebig, J. ... ... *Animal Chemistry.* (Ed. by W. Gregory) ... (2) 1842

Liston, R. ... ... *Elements of Surgery* ... ... ... ... (2) 2nd Ed. 1840

Mackenzie, Sir G. S. *Illustrations of Phrenology* ... ... ... ... ... (2) 1820

Martinet, L. ... ... *Pathology.* (Tr. by J. Quain) ... ... ... ... (2) 1826

" ... ... *Manual of Therapeutics.* (Tr. by R. Norton) (2) 2nd Ed. 1832

**Mauriceau, F.** ... *The Diseases of Women with Child, And in Child-bed.* (Tr. by H. Chamberlen) ... ... (2) 8th Ed. 1752
**Mayo, H.** ... ... *Outlines of Human Physiology* ... ... (2) 2nd Ed. 1829
**Miller, J.** ... ... *Principles of Surgery* ... ... ... ... ... ... (2) 1844
,, ... ... *Practice of Surgery* ... ... ... ... ... ... (2) 1846
*Miscellanea Curiosa Medico-physica.* Vols. IV.—VII. ... ... ... (1) 1676–77
**Montgomery, W. F.** *The Signs and Symptoms of Pregnancy* ... ... (2) 1837
**Morton, T.** ... ... *The Surgical Anatomy of the Perinæum* ... ... (2) 1838
**Müller, J.** ... ... *Embryology, with the Physiology of Generation.* (Tr. by W. Baly) ... ... ... ... ... ... (2) 1848
**Murray, J.** ... ... *Elements of Chemistry.* 2 vols. in 1 ... ... (2) 1801

**Neubauer and J. Vogel, C.** *On the Urine.* 4th Ed. (Tr. by W. O. Markham) ... ... ... ... ... ... ... (2) 1863
**Nicholl, W.** ... ... *General Elements of Pathology* ... ... ... ... (2) 1820
**Nisbet, W.** ... ... *The Clinical Guide.* Part III., *Midwifery and Obstetrical Pharmacy* ... ... ... ... ... (2) 1800
**Norton, A. T.**... ... *Osteology for Students* and *Atlas of Plates* ... (2) 1866

**Ormerod, W. P.** ... *Clinical Collections in Surgery* ... ... ... ... (2) 1846

**Paris, J. A.** ... ... *A Treatise on Diet* ... ... .. ... (2) 5th Ed. 1837
**Parker, L.** ... ... *Treatment of Secondary Syphilis* ... ... ... (2) 1850
**Parkes, S.** ... ... *The Chemical Catechism* ... ... ... (4) 5th Ed. 1811
*Patent* alias *Quack Medicines.* 2 vols.. ... ... ... ... ... ... ... ... ... [N.D.]
*Pharmacopœia Collegii Regalis Medicorum Londiniensis* ... ... ... ... (2) 1824
*Pharmacopœia, Edinburgh* ... ... ... ... ... ... ... ... ... (2) 2nd Ed. 1841
**Porter, W. H.** ... *On the Surgical Pathology of the Larynx and Trachea* (2) 1837

**Quain, J.** ... ... *Anatomy.* 10th Ed. (Ed. by E. A. Schäfer and G. D. Thane.) Vol. III., Part I. ... ... ... 1893

**Radicke, —.** ... ... *The Application of Statistics to Medical Enquiries.* (Tr. by F. T. Bond) ... ... ... ... ... (2) 1861
**Rhazes, —.** ... ... *On the Smallpox and Measles.* (Tr. by W. A. Greenhill)... ... ... ... ... ... ... ... (2) 1848
**Richeraud, A.** ... *Elements of Physiology.* (Tr. by G. M. de Lys) (2) 5th Ed. 1812
**Rogers, J.** ... ... *Autobiography of a Workhouse Medical Officer.* (Ed. by T. Rogers) ... ... ... ... ... ... ... (5) 1889
**Rosenstein, N. R.** v. *The Diseases of Children, and their Remedies.* (Tr. by A. Sparrman) ... ... .. ... ... ... (2) 1766
**Ross and J. S. Bury, J.** *A Treatise on Peripheral Neuritis* ... ... ... ... 1893
**Rucco, J.** ... ... *The Science of the Pulse.* 2 vols. ... ... ... (2) 1827
**Ryan, M.** ... ... *Manual of Midwifery* ... ... ... ... (2) 3rd Ed. 1831

**Scarpa, A.** ... ... *Practical Observations on the Principal Diseases of the Eye.* (Tr. by J. Briggs).. ... ... ... (2) 1806
**Schleiden, T. Schwann and M. J.** *Researches.* (Tr. by H. Smith)... (2) 1847
**Schofield, A. T.** ... *Elementary Physiology for Students* ... ... ... ... 1892

**Schroeder van der Kolk, J. L. C.** *On the Spinal Cord.* (Tr. by W. D. Moore) ... ... ... ... ... ... ... ... ... (2) 1859

" " *Atrophy of the Brain.* (Tr. by W. D. Moore) ... ... ... ... ... ... ... ... ... (2) 1861

**Schwann and M. J. Schleiden, T.** *Researches.* (Tr. by H. Smith)... (2) 1847

**Scratchley, J.** ... *The London Dissector* ... ... ... ... ... (2) 7th Ed. 1826

**Senator, H.** ... ... *Albuminuria in Health and Disease.* (Tr. by T. P. Smith) ... ... ... ... ... ... ... ... ... ... 1884

**Siebold, C. T. v.** ... *On Worms.* (Tr. by T. H. Huxley) ... ... ... 1857

**Skey, F. C.** ... ... *Operative Surgery* ... ... ... ... ... ... ... ... (2) 1850

**Smellie, W.** ... ... *A Treatise on the Theory and Practice of Midwifery.* (2) Vol. I., 5th Ed., 1766; (2) Vol. II., 4th Ed., 1768; (2) Vol. III., 3rd Ed. ... ... 1772

**Smith, N.** ... ... *The More Severe Forms of Lateral Curvature of the Spine* ... ... ... ... ... ... ... ... ... ... ... 1892

**South, J. F.** ... ... *A Description of the Bones* ... ... ... (2) 2nd Ed. 1828

**Stevens, A. A.** ... *A Manual of the Practice of Medicine* ... ... ... 1893

**Stewart, A. P.** ... *On the Nature and Pathologv of Typhus and Typhoid Fever.* (Ed. by W. Cayley) ... ... ... ... 1884

*Surgeon's Vade-Mecum, The* ... ... ... ... ... ... ... ... ... (2) 1809

*Surgery, Memoirs of the Academy of.* (Tr. and Ed. by D. Ottley) ... (2) 1848

**Taylor, A. S.** ... ... *Medical Jurisprudence.* ... ... ... (2) 2nd Ed. 1846

**Tenner and A. Kussmaul, A.** *On Epileptiform Convulsions from Hæmorrhage.* (Tr. by E. Bronner) ... ... (2) 1859

**Thomson, T.** ... ... *A System of Chemistry.* 4 vols. ... (2) 5th Ed. 1817

**Vogel, J.** ... ... *Pathological Anatomy of the Human Body.* (Tr. by G. E. Day) ... ... ... ... ... ... ... ... (2) 1847

**Vogel and C. Neubauer, J.** *On the Urine.* 4th Ed. (Tr. by W. O. Markham) ... ... ... ... ... ... ... ... (2) 1863

**Walker, J.** ... ... *Oculist's Vade-mecum* ... ... ... ... ... ... ... (2) 1843

**Walker, J. K.** ... *On the Peculiarities of Diseases of Children* ... (2) 1835

**West, C.** ... ... ... *Diseases of Infancy and Childhood* ... ... ... ... 1848

**White, R.** ... ... *Practical Surgery* ... ... ... ... ... ... (2) 2nd Ed. 1796

**Whitelegge, B. A.**... *Hygiene and Public Health*.... ... ... ... 2nd Ed. 1893

---

### TRANSACTIONS, REPORTS, JOURNALS, &c.

*The List of current Periodicals to be seen in the Library is given in another part of this number.*

American Journal of Obstetrics, The ... ... ... ... Vol. XXVI. 1892

American Journal of the Medical Sciences, The ... ... Vol. CIV. 1892

American Surgical Association, Transactions of the ... ... Vol. X. 1892

Archives de Neurologie ... ... ... ... ... ... ... ...Tome XXIV. 1892

Birmingham Medical Review, The ... ... ... ... ...Vol. XXXII. 1892

Bristol Medico-Chirurgical Journal ... ... ... ... ... ... Vol. X. 1892

British Journal of Dermatology, The ... ... ... ... ... Vol. IV. 1892
British Medical Journal ... ... ... ... ... ... ... ... Vol. II. 1892
Calendar of the Royal College of Surgeons of England, The
(2) 1869; 1874-78
Cincinnati Lancet-Clinic, The ... ... ... ... ... ... Vol. LXVIII. 189
Deutsche medicinische Wochenschrift ... ... ... ... ... ... ... 1891-92
Dublin Journal of Medical Science, The ... ... ... .. Vol. XCIV. 1892
Fortschritte der Krankenpflege ... ... ... ... ... ... ... ... ... ... 1892
Gazette de Gynécologie ... ... ... ... ... ... ... Tome VII. 1892
Gazette des Hôpitaux ... ... ... ... ... ... ... ... ... ... ... ... 1892
Glasgow Medical Journal, The ... ... ... ... ... Vol. XXXVIII. 1892
Hospital Annual, Burdett's ... ... ... ... ... ... ... ... .. 1893
Hospital, The ... ... ... ... ... ... ... ... ... ... ... Vol. XII. 1892
Hospitals—
Johns Hopkins Hospital Reports. Vol. III., Nos. 1, 2, 3 ... 1892
Middlesex Hospital. Registrars' Reports ... ... ... ... ... ... 1892
Saint Bartholomew's Hospital Reports ... Vol. XXVIII. 1892
Journal of Cutaneous and Genito-Urinary Diseases ... ... Vol. X. 1892
Journal of Laryngology, Rhinology, and Otology, The ... Vol. VI. 1892
Journal of Microscopy, The ... ... ... ... ... ... ... Vol. XI. 1892
Journal of Nervous and Mental Disease, The N.S. ... Vol. XVI. 1892
Journal of the American Medical Association, The ... Vol. XIX. 1892
Lancet, The. (2) Vol. I., 1828; (2) Vol. II., 1838-39; (2) Vol. II., 1841; (2) 1849; (2) Vol. II., 1852; (2) Vol. II., 1853; ... Vol. II. 1892
London Medical and Surgical Journal, The ... ... Vols. II.—V. (2) 1833-34
Medical Age, The ... ... ... ... ... ... ... ... ... ... ... Vol. X. 1892
Medical Annual, The ... ... ... ... ... ... ... ... ... ... ... ... 1893
Medical Chronicle, The ... ... ... ... ... ... ... Vol. XVI. 1892
Medical Circular, The ... ... ... ... ... ... ... ... (2) Vol. IV. 1854
Medical Directory, The ... ... ... ... ... ... ... ... ... ... ... 1893
Medical Record ... ... ... ... ... ... ... ... ... Vol. XLII. 1892
Medical Review ... ... ... ... ... ... ... ... ... Vol. XXVI. 1892
Medico-Chirurgical Review, The .. ... ... ... (2) Vols. I.—IV. 1821-24
Medico-Chirurgical Transactions. (6) Vol. I., 2nd Ed., 1812; (6) Vols. II.—VII., 1811-16; (6) Vol. VIII., Part II.—Vol. XII., Part I., 1817-22; (6) Vol. XIII., Part II., 1827; (6) Vol. XIV., Part I., 1828; (6) Vols. XV.—XVI., Part I., 1829-30; (6) Vols. XIX.—XXXII., 1835-49; (6) Vol. LXVII. ... ... ... ... ... 1884
New York Medical Journal, The ... ... ... ... ... Vol. LVI. 1892
New Zealand Medical Jonrnal, The ... ... ... ... ... Vol. III. 1890
Retrospect of Medicine, Braithwaite's ... ... ... ... Vol. CVI. 1893
Revue de Laryngologie, d'Otologie et de Rhinologie ... Tome XII. 1892
Royal Academy of Medicine in Ireland, Transactions of the Vol. X. 1892
Union médicale, L' ... ... ... ... ... ... ... ... ... Tome LIV. 1892
Year-Book of Medicine and Surgery, A ... ... ... ... (2) 4 vols. 1860-63
Year-Book of Treatment, The ... ... ... ... ... ... ... ... ... 1893

---

In Vol. X., p. 335, for Graves, E. J., read Graves, R. J.; and p. 337, line 3, for 1891 read 1871.

### Bristol Medico-Chirurgical Society.

### President.

E. MARKHAM SKERRITT, M.D., B.S., B.A. Lond.

### President-Elect.

J. GREIG SMITH, M.B., C.M., M.A. Aberd., F.R.S.E.

### Honorary Secretary.

G. MUNRO SMITH.

### Committee.

*Past Presidents.*
R. W. COE.
AUGUSTIN PRICHARD, M.D. Berlin.
J. G. SWAYNE, M.D. Lond.
E. LONG FOX, M.D. Oxon.
JAMES G. DAVEY, M.D. St. And.
W. JOHNSTONE FYFFE, M.D. Dub.
W. H. SPENCER, M.D., M.A. Cantab.
F. POOLE LANSDOWN.
L. M. GRIFFITHS.
R. SHINGLETON SMITH, M.D., B.Sc. Lond.
NELSON C. DOBSON.
S. H. SWAYNE.
F. R. CROSS, M.B. Lond.

J. MICHELL CLARKE, M.D., M.A. Cantab.
JOHN DACRE.
A. J. HARRISON, M.B. Lond.
W. H. HARSANT.
ARTHUR W. PRICHARD.
J. E. SHAW, M.B., C.M. Ed.

Medical Men wishing to join the Society are requested to communicate with the Honorary Secretary, G. MUNRO SMITH, 28 Alma Road, Clifton, Bristol. The Annual Subscription is 21/-.

---

*Extracts from Journal By-laws.*

4.—The *Journal* shall be delivered free to Subscribers and also to Members of the Society whose subscriptions are not in arrear.

6.—The Annual Subscription to the *Journal* for those who pay before the 1st of July shall be 5/-, post free, or 6/- if paid after that date.

---

Communications intended for insertion in the *Journal* should be sent to the Editor, Dr. SHINGLETON SMITH, Deepholm, Clifton Park, Clifton, Bristol.

Books for Review, Exchange Journals, and communications referring to the delivery of the *Journal* should be sent to the Assistant-Editor, L. M. GRIFFITHS, 9 Gordon Road, Clifton, Bristol, to whom Subscriptions are to be paid *in advance.*

Authors requiring Reprints of their Papers should communicate with the Publisher.

Cases for Binding Vols. I.–X. are now ready, and may be obtained, price 7*d.* each (postage 2*d.* extra), upon application to J. W. ARROWSMITH, Quay Street, Bristol; or the *Journal* will be bound, including Case, for 1/3 per volume.

### Bristol Medico-Chirurgical Society.

---

### PAST PRESIDENTS.

1874—75. FREDERICK BRITTAN, M.D., B.A. Dub.

1875—76. ROBERT WILLIAM COE.

1876—77. SAMUEL MARTYN, M.D. Ed.

1877—78. AUGUSTIN PRICHARD, M.D. Berlin.

1878—79. HENRY EDWARD FRIPP, M.D. Würzburg.

1879—80. WILLIAM MICHELL CLARKE.

1880—81. JOSEPH GRIFFITHS SWAYNE, M.D. Lond.

1881—82. EDWARD LONG FOX, M.D. Oxon.

1882—83. JAMES GEORGE DAVEY, M.D. St. And.

1883—84. WILLIAM JOHNSTONE FYFFE, M.D. Dub

1884—85. GEORGE FORSTER BURDER, M.D. Aberd.

1885—86. WILLIAM HENRY SPENCER, M.D., M.A. Cantab

1886—87. FRANCIS POOLE LANSDOWN.

1887—88. LEMUEL MATTHEWS GRIFFITHS.

1888—89. ROBERT SHINGLETON SMITH, M.D., B.Sc. Lond

1889—90. NELSON CONGREVE DOBSON.

1890—91. SAMUEL HENRY SWAYNE.

1891—92. FRANCIS RICHARDSON CROSS, M.B. Lond.

1893.

### LIST OF SUBSCRIBERS

TO THE

### Bristol Medico-Chirurgical Journal.

*Those marked * are Members of the Society.*

| | |
|---|---|
| Ackland, J. McKno | 24 Southernhay, Exeter. |
| *Ackland, W. R. | 5 Rodney Place, Clifton. |
| Adams, George | 11 Westbourne Place, Clifton. |
| *Adams, John Barfield | 110 Cotham Road, Bristol. |
| Alexander, James, M.D., M.Ch., B.A. Dub. | Bishop's Place, Paignton. |
| Aldridge, Charles, M.D., C.M. Aberd. | Plympton. |
| Aldous, George F. | Compton Gifford, Plymouth. |
| *Alford, George Ernest | 5 Royal Crescent, Weston-super-Mare |
| Alford, Henry J., M.D. Lond. | 1 Hovelands Terrace, Taunton. |
| Anstie, Thomas B. | 21 Northgate Street, Devizes. |
| Arthur, John | Hilton, Llandaff. |
| *Atchley, George F., M.B. Lond. | Tyndall's Park, Bristol. |
| Avarne, A. B. | Blaenavon, Monmouthshire. |
| *Aveline, H. T. S. | Asylum, Stapleton, Gloucestershire. |
| Baker, A. de W. | 2 Lawn Terrace, Dawlish. |
| Ballantyne, J. W., M.D., C.M. Ed. | 24 Melville Street, Edinburgh. |
| Bannatyne, Gilbert A., M.D., C.M. Glasg. | 1 Paragon, Bath. |
| *Barclay, W. M. | Queen's Road, Clifton. |
| Barker, Arthur E. | 87 Harley Street. London, W. |
| Baretti, T. G. L'Enardi | 49 Royal York Crescent, Clifton. |
| *Baron, Barclay J., M.B., C.M. Ed. | 16 White Ladies Road, Clifton. |
| Barr, J. Coleman | 2 Cranmore Cottages, Aldershot. |
| *Basset, Walter | 24 Stow Hill, Newport, Mon. |
| *Bateman, F. J. B. | Alburgh House, Wells, Somersetshire |
| Batten, Rayner W., M.D. Lond. | 1 Brunswick Square, Gloucester. |
| Batten, W. Smith | Curzon House, Calne. |
| *Beddoe, John, M.D. Ed., B.A. Lond., F.R.S. | The Chantry, Bradford-on-Avon. |
| Benham, Harry A., M.D., C.M. Aberd. | Asylum, Stapleton, Gloucestershire. |
| Bennett, W. S. | Escot, Penzance. |
| *Benson, A. H. | Wrington, Somersetshire. |
| Berry, James, M.B., B.S. Lond. | 60 Welbeck Street, London, W. |
| *Blacker, A. E. | Richmond Hill Avenue, Clifton. |
| *Blacker, E. | 12 White Ladies Road, Clifton. |
| *Blagg, Arthur F. | 6 West Mall, Clifton. |
| Blomfield, Arthur G., M.D. Aberd. | 34 West Southernhay, Exeter. |
| Bloxam, G. E. | Cambridge House, Lower Weston, Bath. |
| *Board, Edmund C. | 11 Caledonia Place, Clifton. |
| *Boyce, James G. | 6 Colston Parade, Bristol. |
| Boys, Arthur Henry | Chequer Street, St. Alban's. |

### LIST OF SUBSCRIBERS.

| | |
|---|---|
| Brabazon, A. B., M.D. Aberd. | 12 Darlington Street, Bath. |
| Bright, J. A. | Glastonbury. |
| Brook, W. F. | Mount Pleasant, Swansea. |
| *Broom, John, M.D., Ch.D., Obst.D. Brussels | St. Paul's Road, Clifton. |
| Brown, G. Arthur | Tredegar. |
| Brown, William, M.D., C.M. Glasg. | Fishponds, Gloucestershire. |
| Browne, A. E. Newbury | Burbage, Wiltshire. |
| Browne, G. Henry | The Hermitage, Brynmawr, Breconshire. |
| Browne, J. Walton, M.D., B.A. Roy. Univ. I. | 10 College Square North, Belfast. |
| Browne, Lennox | 15 Mansfield Street, London, W. |
| Burges, R. E., M.D., M.Ch., B.A. Roy. Univ. I. | 3 Egerton Terrace, Hoole Road, Chester. |
| Burroughs, E. F. H. | 3 Elgin Park, Bristol. |
| *Bush, J. Paul | Clifton Park, Clifton. |
| | |
| *Cameron, John, M.B., C.M. Glasg. | Ashley Hill, Bristol. |
| Campbell, George | Chilton Polden. |
| Carolin, George | Stonehouse, Gloucestershire. |
| *Carr, Alexander | Wells Road, Bristol. |
| Carter, R. W., M.D. Calcutta | 13 Gloucester Row, Weymouth. |
| *Carter, T. M. | 13 Dowry Square, Clifton. |
| *Carter, W. F. | 48 Coronation Road, Bristol. |
| Cave, Edward J., M.D. Lond. | Crewkerne. |
| Cheves, Alexander B., M.D., M.A. Aberd. | Millbrook, Devonshire. |
| *Clarke, John Michell, M.D., M.A. Cantab. | 28 Pembroke Road, Clifton. |
| Clay, Challoner | Manor House, Fovant. |
| Clay, R. Hogarth, M.D. Ed. | 4 Windsor Villas, Plymouth. |
| Close, John B., M.B. Dur. | 61 Spring Bank, Hull. |
| Coates, Harcourt | 17 Endless Street, Salisbury. |
| Coathupe, Edwin W. | 7 Clifton Park, Clifton. |
| Cobbold, C. S. W., M.D. Würzburg | Bailbrook House, Bath. |
| *Coe, R. W. | 7 Pembroke Road, Clifton. |
| Coker, T. V. | 2 Chesterfield Place, Clifton. |
| Cole, Thomas, M.D. Lond. | 18 Circus, Bath. |
| *Collins, H. W. | Wrington, Somersetshire. |
| Colman, Henry | Pewsey, Wiltshire. |
| Colman, Thomas J., M.D. Ed. | Elmgrove Road, Bristol. |
| Colmer, Ptolemy S. H., M.D. Dur. | South Street House, Yeovil. |
| *Cook, Henri, M.B., C.M. Aberd. | 45 Stackpool Road, Bristol. |
| Cooke, A. S. | Badbrook House, Stroud. |
| *Corbould, George G. | 22 Berkeley Square, Bristol. |
| Cossham, W. R., M.D., C.M. Aberd. | Cirencester. |
| *Cotton, William, M.B., C.M., M.A. Ed. | 227 Gloucester Road, Bristol. |
| Cousins, J. Ward, M.D. Lond. | Kent Road, Southsea. |
| Cowan, F. S. | 27 Queen Square, Bath. |
| Cox, E. Owen | Gillingham, Dorsetshire. |
| Craddock, Samuel | South Lyncombe, Bath. |
| Crespi, Alfred J. H. | Poole Road, Wimborne. |
| *Cross, F. Richardson, M.B. Lond. | College Road, Clifton. |
| *Crossman, Edward, M.D. Dur. | Hambrook, Gloucestershire. |
| Crouch, C. Percival, M.B. Lond. | 3 Princes Buildings, Weston-super-Mare. |
| | |
| *Dacre, John | St. Paul's Road, Clifton. |
| Daniel, T. P. | Beaminster, Dorsetshire. |
| *Davey, James G., M.D. St. And. | 31 Avonmore Road, London, W. |
| *Davies, D. S., M.D. Lond. | 60 Oakfield Road, Clifton. |
| Davies, Ebenezer | Brunswick House, Swansea. |
| Davies, H. N. | Porth, Glamorganshire. |
| Davis, Robert | Oakleigh, Epsom. |
| Davis, Theodore, M.D. Lond. | Beachcroft, Clevedon. |

### LIST OF SUBSCRIBERS.

| | |
|---|---|
| Davy, Henry, M.D. Lond.... ... ... ... ... | Southernhay House, Exeter. |
| Day, Percy Howard ... ... ... ... ... ... | Stalmine, Poulton-le-Fylde. |
| Deas, P. Maury, M.B., M.S. Lond. ... ... | Wonford House, Exeter. |
| Dening, Edwin ... ... ... ... ... ... ... | Manor House, Stow-on-the-Wold |
| *Devis, Harry F. ... ... ... ... ... ... ... | Wells Road, Bristol. |
| Dew, Henry R. ... ... ... ... ... ... ... | 25 Berkeley Square, Bristol. |
| Dickie, James Steel, M.D., C.M. Aberd. ... | Christchurch Road, Bournemouth. |
| *Dobson, Nelson C. ... ... ... ... ... ... | 27 Victoria Square, Clifton. |
| Domville, Edward J. ... ... ... ... ... ... | 44 Southernhay, Exeter. |
| *Dowson, W., M.B., B.C., M.A. Cantab. ... ... | Great George Street, Bristol. |
| Doyne, Robert W. ... ... ... ... ... ... | 70 Woodstock Road, Oxford. |
| Duffett, E. D. ... ... ... ... ... ... ... | 5 London Street, Swindon, Wiltshire. |
| Duke, Alexander ... ... ... ... ... ... ... | Alconbury, Cheltenham. |
| Dunbar, Eliza L. Walker, M.D. Zürich ... | 9 Oakfield Road, Clifton. |
| *Duncan, William ... ... ... ... ... ... ... | Westbury-on-Trym. |
| | |
| Eadon, W. F. Bailey ... ... ... ... ... ... | Hambrook, Gloucestershire. |
| *Eager, Reginald, M.D. Lond. ... ... ... | Northwoods, Winterbourne. |
| Eddowes, Charles ... ... ... ... ... ... | Maddington, Devizes. |
| *Edgeworth, F. H., M.B., B.C., B.A. Cantab., B.Sc. Lond. ... ... ... ... ... ... ... ... | 1 Richmond Hill, Clifton. |
| Edis, Thomas ... ... ... ... ... ... ... ... | 66 Barton Street, Gloucester. |
| Edwards, W. T., M.D. Lond. ... ... ... ... | 129 Queen Street, Cardiff. |
| Elder, William ... ... ... ... ... ... ... | 7 Trelawney Road, Bristol. |
| *Elliott, Christopher, M.D., B.A. Dub. ... | 88 Pembroke Road, Clifton. |
| Ellis, W. McD., M.D. Brussels ... ... ... | 7 Alexander Buildings, Bath. |
| Elsworth, R. C., M.B., C.M. Ed. ... ... ... | 203 St. Helen's Road, Swansea. |
| Embleton, Dennis C. ... ... ... ... ... ... | St. Michael's Road, Bournemouth. |
| *Etches, W. R. ... ... ... ... ... ... ... | Macclesfield. |
| Evans, J. Fenton, M.B., C.M. Ed. ... ... | 6 Whitehall Yard, London, S.W |
| Evans, W. G. ... ... ... ... ... ... ... ... | Beckington. |
| *Ewens, John ... ... ... ... ... ... ... ... | 14 White Ladies Road, Clifton. |
| | |
| Fairbanks, W., M.D., C.M. Ed. ... ... ... | Wells, Somersetshire. |
| *Fendick, R. George ... ... ... ... ... ... | St. Paul's Road, Clifton. |
| Finch, Thomas, M.B., B.A. Cantab.... ... ... | Watcombe View, Babbacombe. |
| Finch, Thomas, M.D. St. And. ... ... ... | Westville, St. Mary Church. |
| Fisher, Fred. B. ... ... ... ... ... ... ... | West Walk, Dorchester. |
| Flemming, C. E. S. ... ... ... ... ... ... | Freshford. |
| Ford, Joseph ... ... ... ... ... ... ... ... | Wedmore. |
| Forty, D. H. ... ... ... ... ... ... ... ... | Wotton-under-Edge. |
| Fox, Arthur E. W., M.B., C.M. Ed. ... ... | 16 Gay Street, Bath. |
| *Fox, Bonville B., M.D., M.A. Oxon. ... ... | Brislington. |
| Fox, Charles Henry, M.D. St. And. ... ... | Brislington. |
| Fox, E. Lawrence, M.D., B.Ch., M.A. Cantab. | 19 Lockyer Street, Plymouth. |
| *Fox, E. Long, M.D. Oxon. ... ... ... ... | Church House, Clifton. |
| Fox, John Alfred... ... ... ... ... ... ... | Morrab Road, Penzance. |
| Fraser, Græme B. ... ... ... ... ... ... | Beach Road, Weston-super-Mare. |
| Freeman, Henry W. ... ... ... ... ... ... | 24 Circus, Bath. |
| Fry, J. Blount ... ... ... ... ... ... ... | Ashley Lodge, Esher. |
| *Fyffe, W. Johnstone, M.D.,. B.A. Dub. ... | 2 Rodney Place, Clifton. |
| | |
| Gamble, C. H.... ... ... ... ... ... ... ... | Barnstaple. |
| George, R. Julyan, M.B., C.M. Ed. ... ... | Port Isaac, Cornwall. |
| *Gibbs, Alfred N. Godby ... ... ... ... ... | 27 Chandos Road, Bristol. |
| Gill, John, M.D. Brussels ... ... ... ... ... | 31 Apsley Road, Clifton. |
| Gloag, G. A. ... ... ... ... ... ... ... ... | 7 College Green, Bristol. |
| Goodden, Wyndham C. ... ... ... ... ... | 14 Berkeley Square, Bristol. |

### LIST OF SUBSCRIBERS.

| | |
|---|---|
| Goodfellow, W. R. | Roche, Cornwall. |
| Goodridge, Henry F. A., M.D. Lond. | 10 Brock Street, Bath. |
| Gordon, W., M.B., B.C., M.A. Cantab. | Barnfield Lodge, Exeter. |
| Goss, T. Biddulph | 1 Circus, Bath. |
| Gould, A. Pearce, M.B., M.S. Lond. | 10 Queen Anne Street, London, W. |
| Grace, Alfred | Chipping Sodbury. |
| Grace, Edward Mills | Thornbury, Gloucestershire. |
| Grace, Henry | Kingswood, Bristol. |
| Grace, W. G. | 15 Victoria Square, Clifton. |
| Granville, J. Mortimer, M.D. St. And. | 14 Hanover Square, London, W. |
| Gratte, C. Brooke | 3 Lansdowne Place, Newport, Mon. |
| Gray, A. Murray | 17 Market Place, Devizes. |
| Grey, T. C. | The Shrubbery, Weston-super-Mare. |
| *Griffiths, C. A. | 190 Easton Road, Bristol. |
| *Griffiths, J. S. | 18 Hampton Road, Bristol. |
| *Griffiths, L. M. | 9 Gordon Road, Clifton. |
| Grote, G. W., M.B. Toronto | 54 Terrace Road, London, E. |
| Groves, J., M.B., B.A. Lond. | Carisbrooke. |
| Guinness, T. A. | Bampton, Devonshire. |
| | |
| *Hailes, Clements D. G., M.D., C.M. Ed. | 9 King's Parade, Clifton. |
| *Haines, A. H. | 8 Cotham Road, Bristol. |
| Hale, Edmund T. | The Grange, Chew Magna. |
| Halpin, W. C. | Parkend. |
| Hamlen-Williams, T. R. | Verlands, Cowbridge. |
| Handfield-Jones, Montagu, M.D. Lond. | 35 Cavendish Square, London, W. |
| Hardwick, A., M.B. Dur. | Newquay, Cornwall. |
| Harper, Joseph | Bear Street, Barnstaple. |
| *Harrison, A. J., M.B. Lond. | Guthrie Road, Clifton. |
| Harrison, Charles | Keynsham, Somersetshire. |
| Harsant, J. G., M.D., B.S. Lond. | Exeter Road, Bournemouth. |
| *Harsant, W. H. | 16 Pembroke Road, Clifton. |
| *Hawkins-Ambler, G. A. | 162 Upper Parliament Street, Liverpool. |
| Hawkins, Cæsar F. | North Petherton. |
| Haydon, Edgar, M.B., C.M. Glasg. | The Laurels, Newton Abbot. |
| Hayes, H. W. McCaulty | 1 Brunswick Terrace, Swindon, Wiltshire. |
| Hayes, W. A. | Elm Grove, Calne. |
| Hayman, Alfred G. | 1 Pembroke Road, Clifton. |
| *Hayman, C. A. | 17 Victoria Square, Clifton. |
| Heane, W. C. | Cinderford. |
| Hearder, G. J., M.D. St. And. | Joint Counties Asylum, Carmarthen. |
| *Heaven, John C. | 2 Queen Square, Bristol. |
| *Herapath, C. K. C. | Cotham Park, Bristol. |
| *Hetling, H. E. | Stapleton Road, Bristol. |
| Hill, Hedley | 1 Redcliff Hill, Bristol. |
| Hills, Frederic F. | 164 Stapleton Road, Bristol. |
| Hoblyn, Francis Parker | 10 Bladud Buildings, Bath. |
| *Hoffman, A. W. W. | 25 Gordon Road, Clifton. |
| Hopkins, H. Culliford | 4 Gay Street, Bath. |
| Horsfall, John, M.A. Oxon. | Bath Road, Bournemouth. |
| Hospital, Bristol General (*Sec.*, W. Thwaites). | |
| Hospital, Taunton and Somerset (*House-Surgeon*, J. G. Bain). | |
| Howells, William, M.B., C.M. Glasg. | Church House, Talgarth, Breconshire. |
| Howse, William | 8 London Street, Swindon, Wiltshire. |
| Howsin, E. Arthur, M.D. St. And. | Stroud. |
| Hudson, F. Horace | Sturminster Newton. |
| Hudson, H. R. | 182 Commercial Road, Newport, Mon. |
| Huggard, W. R., M.D., M.Ch., M.A. Roy. Univ. I. | Davos-Platz, Switzerland. |

### LIST OF SUBSCRIBERS.

| | |
|---|---|
| Hunt A. D. ... ... ... ... ... ... ... ... | Millbrook House, Chagford. |
| Hyatt, J. T. ... ... ... ... ... ... ... ... | Shepton Mallet. |

| | |
|---|---|
| *Imlay, G. A., M.B., C.M. Aberd. ... ... ... | 1 Cotham Park, Bristol. |
| Infirmary, Bristol Royal (*Librarian*, Walter J. Hill). | |
| Institution, Liverpool Medical (*Resident Librarian*, William Jones). | |
| Irwin, Alan M. ... ... ... ... ... ... ... | Blakeney, Gloucestershire. |
| Ives, William R. Y. ... ... ... ... ... ... | Portswood Road, Southampton. |

| | |
|---|---|
| Jackson, J. B., M.D., M.Ch. Roy. Univ. I. ... | New York, U.S.A. |
| Jackson, Mark, M.D. Roy. Univ. I.... ... ... | Bear Street, Barnstaple. |
| Jacobson, W. H. A., M.B., M.C., M.A. Oxon. | 66 Great Cumberland Place, London, W. |
| James, J. Bowen ... ... ... ... ... ... ... | Woodlands, Aberaman, Aberdare. |
| Jessop, Charles Moore ... ... ... ... ... ... | 98 Sutherland Avenue, London, W. |
| *Johnston, D. G., M.B., C.M. Glasg. ... ... | 92 Hampton Road, Bristol. |
| Jones, A. Orlando, M.D., C.M. Aberd. ... | Harrogate. |
| Jones, E. R. ... ... ... ... ... ... ... ... ... | Cilgynlle-fawr, New Quay, Cardiganshire. |
| Jones, Evan ... ... ... ... ... ... ... ... ... | Ty-mawr, Aberdare. |
| Jones, H. Macnaughton, M.D., M.Ch. Roy. Univ. I. ... ... ... ... ... ... ... ... ... ... | 141 Harley Street, London, W. |
| Jones, W. R., M.D., C.M. Glasg. ... ... ... | Senny Bridge, Breconshire. |
| Jones, W. W., M.B., C.M. Ed. ... ... ... | 47 Wellington Street, Merthyr Tydvil. |

| | |
|---|---|
| *Kelly, T. B. ... ... ... ... ... ... ... ... ... | Eye Hospital, Bristol. |
| *Kemm, F. St. J.... ... ... ... ... ... ... ... ... | Worle. |
| Kempe, Arthur W., M.D. Brussels ... ... | 14 Southernhay, Exeter. |
| Kendall, Bernard C.... ... ... ... ... ... ... | Elm Grove, Penally, Pembrokeshire. |
| Kent-Jones, D. ... ... ... ... ... ... ... ... | Plascoed, Deri. |
| King, Louis J.... ... ... ... ... ... ... ... ... | 10 Russel Street, Bath. |
| Kinneir, R. ... ... ... ... ... ... ... ... ... | The Tower House, Malmesbury. |
| Kitson, F. P. ... ... ... ... ... ... ... ... ... | Beaminster, Dorsetshire. |

| | |
|---|---|
| Lace, F. ... ... ... ... ... ... ... ... ... ... | 5 Paragon, Bath. |
| Laing, W. A. Gordon, M.D., C.M. Glasg. ... | Boutport Street, Barnstaple. |
| *Lane, Hugh ... ... ... ... ... ... ... ... | 11 Circus, Bath. |
| *Lansdown, F. Poole ... ... ... ... ... ... | 19 White Ladies Road, Clifton. |
| *Lansdown, R. G. P., M.B., B.S. Dur. ... ... | 39 Oakfield Road, Clifton. |
| Lauwers, Dr. .. ... ... ... ... ... ... ... ... | Courtrai, Belgium. |
| *Lawrence, A. E. Aust, M.D., C.M. Aberd. . | 19 Richmond Hill, Clifton. |
| *Lawrence, Henry ... ... ... ... ... ... | 19 Park Row, Bristol. |
| Lawrie, J. Macpherson, M.D., C.M. Glasg. | Greenhill, Weymouth. |
| Laxton, T. L.... ... ... ... ... ... ... ... ... | 4 Park Place, London, S.W. |
| Leach, J. Comyns, M.D. Dur., B.Sc. Lond. . | Sturminster Newton. |
| *Lees, E. Leonard, M.D., C.M. Ed. ... ... | Redland Road, Bristol. |
| Leigh, W. W. ... ... ... ... ... ... ... ... ... | Treharris, Glamorganshire. |
| Lewellyn, John ... ... ... ... ... ... ... ... | Caerphilly. |
| Library, Bristol Museum and ... ... ... | Queen's Road, Bristol. |
| Library, Cheltenham ... ... ... ... ... ... | 5 Royal Crescent, Cheltenham. |
| *Lidderdale, James ... ... ... ... ... ... | Henley-on-Thames. |
| Lilley, G. Herbert, M.D. Brussels ... ... | H.M. Convict Prison, Portland. |
| Limbery, Thomas ... ... ... ... ... ... ... ... | 184 Commercial Road, Newport, Mon. |
| Lister, Sir Joseph, Bart., F.R.S. ... ... ... | 12 Park Crescent, London, N.W. |
| Lloyd, Walter E. ... ... ... ... ... ... ... | 60 York Road, Bristol. |
| Logan, Frederic T. B. ... ... ... ... ... ... | Dean Lane, Bristol. |
| *Lowe, T. Pagan ... ... ... ... ... ... ... ... | 2 Belmont, Bath. |

### LIST OF SUBSCRIBERS.

| | |
|---|---|
| Luckham, L. S. | 16 Harcourt Terrace, Salisbury. |
| Lucy, Reginald H., M.B., C.M. Ed. | 9 Princess Square, Plymouth. |
| | |
| Macdonald, J. A., M.D., M.Ch. Roy. Univ. I. | 19 East Street, Taunton. |
| Macdonald, P. W., M.D., C.M. Aberd. | County Asylum, Dorchester. |
| *Maclean, Ewen J., M.D., C.M. Ed. | Chelsea Hospital for Women, London |
| Maclean, J. Campbell, M.B., C.M Ed. | Swindon House, Swindon, Wiltshire |
| *Macleod, H. W. G., M.B., C.M. Ed. | 23 Victoria Square, Clifton. |
| Macready, J. | 51 Queen Anne Street, London, W. |
| *Maddison, W. T., M.D. Lond. | 172 Stapleton Road, Bristol. |
| Manning, H. J., B.A. Lond. | Laverstock House, Salisbury. |
| Marley, Henry F. | The Nook, Padstow, Cornwall. |
| Marsh, O. E. Bulwer | Ventnor House, Newport, Mon. |
| Marshall, Arthur L., M.B., B.A. Cantab. | 56 Rectory Road, London, N. |
| *Marshall, Henry, M.D. Ed., F.R.S.E. | 28 Caledonia Place, Clifton. |
| *Martin, E. F., M.B. Ed. | Victoria House, Weston-super-Mare. |
| Martin, Theodore | Temple Cloud. |
| Mason, S. B. | 12 Park Terrace, Pontypool. |
| Mason, William | Sedgemoor Cottage, St. Austell. |
| *Matthews, Charles E. | 51 Pembroke Road, Clifton. |
| Mavrojanni, Dr. | Corfu, Greece. |
| Mayor, T. O. | 40 Apsley Road, Clifton. |
| McElfatrick, John | The Chantry, Mere, Wiltshire. |
| McNicoll, John | High Street, Poole. |
| Meredith, John, M.D. Ed. | Wellington, Somersetshire. |
| Metford, J. Seymour | 34 West Mall, Clifton. |
| Milne, John, M.B., C.M. Aberd. | Hill Avenue, Bristol. |
| Milward, James, M.D. Brussels | 54 Charles Street, Cardiff. |
| Mole, Harold F. | Royal Infirmary, Bristol. |
| Mouat-Biggs, C. E. F. | 2 Fauconberg Villas, Cheltenham. |
| Monckton, William | Portishead. |
| Morgan, Frederick | Uffculme. |
| Morgan, John | Langport, Somersetshire. |
| *Morton, C. A. | 24 St. Paul's Road, Clifton. |
| Munden, Charles | Ilminster. |
| Murray, William, M.B., C.M. Ed. | Staple Hill, Gloucestershire. |
| *Myles, G. T. | 3 Portland Square, Bristol. |
| | |
| Napier, T. W. A., M.D., C.M. Aberd. | Church Street, Egremont, Cheshire. |
| Neale, B. G. | Uffculme. |
| Nell, Richard F. | 33 Windsor Terrace, Penarth. |
| *Nevill, W. N., M.D., B.Ch., B.A. Dub. | Southville, Bristol. |
| *Newman, Charles | 156 Cheltenham Road, Bristol. |
| *Newnham, W. H. C., M.B., M.A. Cantab. | St. Paul's Road, Clifton |
| Newstead, James | 9 York Place, Clifton. |
| Nicholson, T. D., M.D., C.M. Ed. | 2 White Ladies Road, Clifton. |
| Norgate, R. H. | County Asylum, Maidstone. |
| Norman, J. H. | Goodleigh, Winkleigh, Devonshire. |
| *Norton, J. A., M.D., C.M. Aberd. | 65 Redcliff Hill, Bristol. |
| | |
| Odell, William | Ferndale, Torquay. |
| *Ogilvy, Alexander, M.D., B.Ch., B.A. Dub. | Eye Hospital, Bristol. |
| Openshaw, E. Hyde | Moreton Hampstead. |
| *Ormerod, Henry | Westbury-on-Trym. |
| | |
| Page, G. Shepley | 78 Old Market Street, Bristol. |
| *Parker, George, M.D., M.A. Cantab. | 14 Pembroke Road, Clifton. |

### LIST OF SUBSCRIBERS.

| | |
|---|---|
| PARRY, J. H. ... ... ... ... ... ... ... ... | 199 Cheltenham Road, Bristol. |
| PARSONS, FRANCIS J. C. ... ... ... ... ... ... | King Square, Bridgwater. |
| PARSONS, FREDERICK ... ... ... ... ... ... | Common Hill, Frome. |
| PATERSON, DONALD ROSE, M.D., C.M. Ed.... | 18 Windsor Place, Cardiff. |
| PAULI, G. C. P. ... ... ... ... ... ... ... | 240 York Road, Bristol. |
| PEAKE, G. ARTHUR ... ... ... ... ... ... ... | Alma House, Cheltenham. |
| *PENNY, F. ... ... ... ... ... ... ... ... ... | North Devon Infirmary, Barnstaple. |
| *PENNY, W. J. ... ... ... ... ... ... ... ... | Richmond Park Road, Clifton. |
| PENTLAND, YOUNG J. ... ... ... ... ... ... | 11 Teviot Place, Edinburgh. |
| PESKETT, A. W. C., M.B., B.C., M.A. Cantab. | Burnham, Somersetshire. |
| PHELPS, W. HARFORD G., M.D. Aberd. ... | Beachfield, Weston-super-Mare. |
| PHILLIPS, EDWARD PICTON ... ... ... ... ... | High Street, Haverfordwest. |
| PHILLIPS, J. A. ... ... ... ... ... ... ... ... | 72 Carlisle Street, Cardiff. |
| *PICKERING, C. F. ... ... ... ... ... ... ... | 12 Berkeley Square, Bristol. |
| PIPPETTE, WALTER... ... ... ... ... ... ... | Dawes Road, London, S.W. |
| PIZEY, G. F. P.... ... ... ... ... ... ... ... | 5 Prospect Terrace, Babbacombe. |
| PLAISTER, W. H. ... ... ... ... ... ... ... | High Road, Tottenham. |
| POLLARD, REGINALD, M.B. Dur. ... ... ... | Southlands, Torquay. |
| *POUNTNEY, W. E., M.D., C.M. Ed. ... ... | 33a White Ladies Road, Bristol. |
| *POWELL, J. J., M.D., Lond. ... ... ... ... | 2 Portland Square, Bristol. |
| POWELL, R. DOUGLAS, M.D. Lond.... ... ... | 62 Wimpole Street, London, W. |
| POWELL, WILLIAM, M.B. Lond. ... ... ... | Hill Garden, Torquay. |
| PRICE, R. G., M.D. Dur.... ... ... ... ... ... | Carmarthen. |
| PRICE, WILLIAM, M.B. Lond. ... ... ... ... | 40 Park Place, Cardiff. |
| *PRICHARD, ARTHUR W.... ... ... ... ... ... | Gordon Road, Clifton. |
| *PRICHARD, AUGUSTIN, M.D. Berlin... ... ... | 4 Chesterfield Place, Clifton. |
| *PRICHARD, J. E., M.B., B.A. Oxon. ... ... | 243 Cheltenham Road, Bristol. |
| PRIDEAUX, T. ENGLEDUE P. ... ... ... ... | Wellington, Somersetshire. |
| *PRING, ARTHUR, B.A. Cantab. ... ... ... ... | 14 Meridian Place, Clifton. |
| PROWSE, ALBERT P. ... ... ... ... ... ... | Yennadon, Yelverton, Devonshire. |
| *PROWSE, ARTHUR B., M.D. Lond. ... ... ... | 5 Lansdown Place, Clifton. |

| | |
|---|---|
| RANDOLPH, CHARLES ... ... ... ... ... ... | St. Michael's, Milverton, Somersetshire. |
| *RATTRAY J. M., M.D., C.M., M.A. Aberd. ... | Frome. |
| REDWOOD, T. HALL, M.D., C.M., M.A. Dur.... | The Lawn, Rhymney, Monmouthshire. |
| RENDALL, JOHN... ... ... ... ... ... ... ... | Forestside, Lymington. |
| REES, ALFRED ... ... ... ... ... ... ... ... | 46 Loudoun Square, Cardiff. |
| RIDDELL, ANDREW W. ... ... ... ... ... ... | St. Mary Bourne. |
| ROBATHAN, G. B. ... ... ... ... ... ... ... | The Grove, Risca. |
| ROBERTSON, ROBERT, M.D., C.M. Ed. ... ... | Belgrave Road, Ventnor. |
| RODERICK, SYDNEY J., M.B., C.M. Ed. ... ... | Llanelly. |
| *ROGERS, B. M. H., M.D., B.Ch., B.A. Oxon.... | 11 York Place, Clifton. |
| *ROSS, R. A. ... ... ... ... ... ... ... ... | Lodway Villa, Pill. |
| *ROSSITER, GEORGE F., M.B. Lond. ... ... | Cairo Lodge, Weston-super-Mare. |
| *ROUÉ, W. BARRETT, M.D., M.S. Dur. ... ... | 2 Buckingham Place, Clifton. |
| ROW, F. EVERARD ... ... ... ... ... ... ... | 9 Tamar Terrace, Devonport. |
| *ROXBURGH, ROBERT, M.B. Ed. ... ... ... ... | 1 Victoria Buildings, Weston-super-Mare |
| *RUDGE, C. KING ... ... ... ... ... ... ... | 145 White Ladies Road, Bristol. |
| *RUDGE, H. T. ... ... ... ... ... ... ... ... | 5 Colston Parade, Bristol. |
| RUFFER, M. ARMAND, M.D., B.S., M.A. Oxon. | 19 Iddesleigh Mansions, London, S.W. |

| | |
|---|---|
| SANDOE, J. W., M.D., B.S. Dur. ... ... ... ... | Broad Clyst. |
| SAWYER, J. A. F. ... ... ... ... ... ... ... | Albert Road, Clevedon. |
| SCIMONELLI, SIGra. CRUCE ... ... ... ... | Naples. |
| SCOTT, JAMES, M.B., C.M. Ed.... ... ... ... | 24 Gaolgate Street, Stafford. |
| SCOTT, RICHARD J. H. ... ... ... ... ... ... | 28 Circus, Bath. |

| | |
|---|---|
| Searle, George C. ... ... ... ... ... ... | Brixham, Devonshire. |
| Searle, Richard Burford ... ... ... ... | St. Just. |
| *Semple, H. F.... ... ... ... ... ... ... ... | Budleigh Salterton, Devonshire. |
| Seward, W. J., M.B. Lond. ... ... ... ... | Asylum, Colney Hatch, London, N. |
| *Shaw, J. E., M.B., C.M. Ed. ... ... ... ... | 23 Caledonia Place, Clifton. |
| Shaw, Jesse, M.D.... ... ... ... ... ... ... | Fort Beaufort, Cape Colony. |
| Sheen, A., M.D. St. And. ... ... ... ... ... | 23 Newport Road, Cardiff. |
| *Sheppard, E. J. ... ... ... ... ... ... ... | 16 Park Place, Clifton. |
| *Shorland, Walter E. ... ... ... ... ... | 189 Cheltenham Road, Bristol. |
| Shortridge, Thomas Wood, M.D. Brussels | Honiton. |
| Simons, C. E. G., M.B., C.M. Aberd. ... ... | The Hollies, Merthyr Tydvil. |
| *Sinclair, David, M.B., C.M. Aberd. ... ... | 8 Osborne Road, Clifton. |
| Sincock, J. Bain ... ... ... ... ... ... ... | Friarn Street, Bridgwater. |
| *Skelton, Henry, M.D. St. And. ... ... ... | Downend, Gloucestershire. |
| *Skerritt, E. Markham, M.D., B.S., B.A. Lond. | Richmond Hill, Clifton. |
| Skinner, George H.... ... ... ... ... ... | Bradford, Victoria, Australia. |
| *Skinner, Stephen, M.B., C.M. Aberd. ... | Tranent Lawn, Clevedon. |
| *Smith, G. Munro ... ... ... ... ... ... ... | 28 Alma Road, Clifton. |
| *Smith, J. Greig, M.B., C.M., M.A. Ab., F.R.S.E. | 16 Victoria Square, Clifton. |
| Smith, Noble ... ... ... ... ... ... ... ... | 24 Queen Anne Street, London, W. |
| Smith, Rev. Reginald, M.A. Oxon.... ... ... | 9 Richmond Hill, Clifton. |
| *Smith, R. Shingleton, M.D., B.Sc. Lond. | Clifton Park, Clifton. |
| Smith, W. Alexander, M.B., M.A. Oxon. ... | Newport, Essex. |
| Snell, Simeon ... ... ... ... ... ... ... | 17 Eyre Street, Sheffield. |
| Society, Cardiff Medical (*Hon. Sec.*, Dr. D. R. Paterson). | |
| Society, Plymouth Medical (*Hon. Lib.*, C. E. R. Rendle). | |
| Society, Royal Medical and Chirurgical | 20 Hanover Square, London, W. |
| *Solly, Reginald V., M.B., B.S. Lond. ... | General Hospital, Bristol. |
| Somer, James ... ... ... ... ... ... ... ... | Broad Clyst. |
| Soutar, James Greig, M.B., C.M. Ed. ... ... | Barnwood House, Gloucester. |
| *Spencer, W. H., M.D., M.A. Cantab. ... ... | 11 Warrior Square, St. Leonard's-on-Sea. |
| Spender, John K., M.D. Lond. ... ... ... | 17 Circus, Bath. |
| Stamp, W. D. ... ... ... ... ... ... ... ... | Ridgeway House, Plympton. |
| Statham, R. Whiteside ... ... ... ... ... | Cheddar, Somersetshire |
| *Steele, Charles, M.D. Dur. ... ... ... ... | 17 Richmond Hill, Clifton. |
| Steele-Perkins, E. B. ... ... ... ... ... | 1 Hill's Buildings, Exeter. |
| *Stevens, W. H. ... ... ... ... ... ... ... | 16 Gloucester Road, Bristol. |
| Stoddart, F. Wallis ... ... ... ... ... ... | 1 Park Street, Bristol. |
| Storry, Frederick W. ... ... ... ... ... | Lansdown, Stroud. |
| *Swain, James, M.D., M.S. Lond. ... ... ... | 14 Buckingham Place, Clifton. |
| *Swayne, J. G., M.D. Lond. ... ... ... ... | 74 Pembroke Road, Clifton. |
| *Swayne, S. H.... ... ... ... ... ... ... ... | 129 Pembroke Road, Clifton. |
| *Swayne, W. Carless, M.B. Lond.... ... ... | St. Paul's Road, Clifton. |
| Swete, H. Lawton... ... ... ... ... ... ... | Fishguard, Pembrokeshire. |
| Sydenham, George F. ... ... ... ... ... | Dulverton, Somersetshire. |
| Symonds, H. P. ... ... ... ... ... ... ... | 35 Beaumont Street, Oxford. |
| Symons, John ... ... ... ... ... ... ... ... | Buriton House, Penzance. |

| | |
|---|---|
| Tait, Lawson ... ... ... ... ... ... ... ... | 7 The Crescent, Birmingham |
| Taylor, C. Bell, M.D. Edin. ... ... ... ... | 9 Park Row, Nottingham. |
| *Taylor, James... ... ... ... ... ... ... | 36 Alma Road, Clifton. |
| Taylor, Thomas Henry ... ... ... ... ... | Thornbury, Gloucestershire. |
| Thomas, A. Garrod, M.D., C.M. Ed. ... ... | Clytha Park, Newport, Mon. |
| Thomas, John T. ... ... ... ... ... ... ... | Chepstow Road, Newport, Mon. |
| Thomas, W. H.... ... ... ... ... ... ... ... | Maesteg, Glamorganshire. |
| Thomson, Eustace B., M.D. C.M. Glasg.... | Albany Place, Plymouth. |
| Thomson, William, M.D., C.M. Ed. ... ... | Mustapha Superieur, Algeria. |
| Thorne, G. Leworthy, M.D. Aberd. ... ... | Cheriton Fitzpaine. |

| | |
|---|---|
| *Tivy, W. J. ... ... ... ... ... ... ... ... ... | 8 Lansdown Place, Clifton. |
| Tomkins, Harding H.... ... ... ... ... ... | 36 Ventnor Villas, Brighton. |
| Torbock, W. H., M.D. Pennsylvania ... ... | Polruan, Cornwall. |
| Tosswill, Louis H., M.B., B.A. Cantab. ... | 28 West Southernhay, Exeter. |
| Tribe, Ethel N., M.B. Lond. ... ... ... ... | 155 White Ladies Road, Bristol. |
| Trotter, Leslie B., M.D., C.M. Ed. ... ... | Coleford, Gloucestershire. |
| Tyley, R. P., M.D. St. And. ... ... ... ... | Wedmore. |
| | |
| Vachell, Herbert R., M.D., C.M. Aberd.... | 15 Newport Road, Cardiff. |
| Vereker, R. H.... ... ... ... ... ... ... ... | Eastleigh, Curry Rivel. |
| | |
| Wade, A. Law, M.D., B.A. Dub. ... ... ... | County Asylum, Wells, Somersetshire. |
| Wade, Charles H., M.A. Oxon. ... ... ... | Stanfield, Torquay. |
| Wade, Reginald ... ... ... ... ... ... ... ... | Highbridge, Somersetshire. |
| *Waldo, Henry, M.D., C.M. Aberd. ... ... | 19 Pembroke Road, Clifton. |
| *Walker, Cyril H., M.B., B.A. Cantab.... ... | St. Paul's Road, Ciflton. |
| Wallace, Rev. Canon, M.A. Oxon. ... ... | 3 Harley Place, Clifton. |
| Walsh, Leslie H. ... ... ... ... ... ... ... ... | Royal United Hospital, Bath. |
| Walter, William Henry, M.D. Brussels ... | Gregory Boulevard, Nottingham. |
| Ward, J. L. W. ... ... ... ... ... ... ... ... | Victoria Street, Merthyr Tydvil. |
| *Wathen, J. Hancocke ... ... ... ... ... ... | 16 York Place, Clifton. |
| Watson, J. Adam ... ... ... ... ... ... ... ... | 39 Dennington Park, London, N.W. |
| Watters, George T. B., M.D., C.M. Ed. ... | Stonehouse, Gloucestershire. |
| Waugh, Alexander ... ... ... ... ... ... ... | Midsomer Norton. |
| Weatherly, Lionel A., M.D., C.M. Aberd. | Bailbrook House, Bath. |
| Webber, William W.... ... ... ... ... ... ... | Crewkerne. |
| Webster, Norman P. ... ... ... ... ... ... ... | George Place, Guernsey. |
| *Webster, Thomas ... ... ... ... ... ... ... | Redland Hill, Bristol. |
| *Wedmore, C. Ernest, M.B., M.A. Cantab. | Stoke Bishop. |
| Wethered, Frank J., M.D. Lond. ... ... | 34 Queen Anne Street, London, W. |
| *Whicher, A. H. ... ... ... ... ... ... ... ... | Midsomer Norton. |
| Whitfeld, W. Clarke ... ... ... ... ... ... | Baggally Street, Hereford. |
| Whyte, Andrew, M.D., C.M. Aberd. ... ... | Honddû House, Brecon. |
| Wickham, W. ... ... ... ... ... ... ... ... ... | Hill House, Tetbury. |
| Wigan, C. A., M.D. Dur. ... ... ... ... ... | Portishead. |
| Wigmore, J., M.D. Roy. Univ. I. ... ... ... | Twerton-on-Avon. |
| Wilcox, Henry, M.B. Aberd. ... ... ... ... | Fleet, Hampshire. |
| *Wilding, James, M.D. Dur. ... ... ... ... | Sydenham Road, Bristol. |
| Willcox, R. Lewis ... ... ... ... ... ... ... | Warminster. |
| Willett, George G. D. ... ... ... ... ... ... | Keynsham, Somersetshire. |
| William, John, M.D. St. And. ... ... ... ... | Brynmeurig, Bethesda. |
| Williams, Charles ... ... ... ... ... ... ... | Howard Gardens, Cardiff. |
| Williams, D. J. ... ... ... ... ... ... ... ... | Greenfield House, Llanelly. |
| Williams, H. Egerton ... ... ... ... ... ... | The Mount, Newport, Mon. |
| *Williams, Lionel H. ... ... ... ... ... ... ... | Thornbury, Gloucestershire. |
| *Williams, P. Watson, M.D. Lond. ... ... | 2 Lansdown Place, Clifton. |
| Williams, Peter ... ... ... ... ... ... ... ... | Ferryside, Carmarthenshire. |
| Wilson, Claude, M.D., C.M. Ed. ... ... ... | Church Road, Tunbridge Wells. |
| *Windsor-Aubrey, H. W. ... ... ... ... ... ... | Coronation Road, Bristol. |
| Winterbotham, L. ... ... ... ... ... ... ... | Bays Hill, Cheltenham. |
| *Wintle, Colston ... ... ... ... ... ... ... ... | Children's Hospital, Bristol. |
| Woodford, E. Russell, M.D., C.M. Ed. ... | 4 Prospect Terrace, Ventnor. |
| Woodhead, G. Sims, M.D., C.M. Ed. ... ... | 1 Nightingale Lane, London, S.W. |
| Woollcombe, Walter L. ... ... ... ... ... ... | 16 Lockyer Street, Plymouth. |
| | |
| Yelf, Leonard K., M.D. St. And. ... ... ... | Moreton-in-Marsh. |

### PUBLICATIONS RECEIVED.

From ADLARD & SON, London: [Reprints].
*Ununited Fractures in Children.* By D'ARCY POWER. 1892. Two papers.

From BAILLIÈRE, TINDALL and COX, London:
*The Hygiene of the Ear.* By Dr. VINCENZO COZZOLINO. Translated by JAMES ERSKINE, M.B. 1892.
*Symptoms and Physical Signs.* By WILLIAM EWART, M.D. 1892.

From BEAUMONT & CO., London:
*Patent* alias *Quack Medicines.* 2 Vols. [N.D.]

From BURROUGHS, WELLCOME & CO., London:
*The A B C Pocket Diary and Memorandum Book.* 1893.
*Helbing's Pharmacological Record.* No. XII. December, 1892.

From T. B. BROWNE, London:

*The Advertiser's A B C.* 1893.

From CASSELL & COMPANY, LIMITED, London:
*Elementary Physiology for Students.* By ALFRED T. SCHOFIELD, M.D. 1892.
*Hygiene and Public Health.* By B. ARTHUR WHITELEGGE, M.D. Second Edition. 1893.

From J. CHARPENTIER, Paris:

*The Medical Week.* Vol. I., No. 3. December 16th, 1892.

From J. & A. CHURCHILL, London:
*Syphilis and the Nervous System.* By W. R. GROVES, M.D. 1892.
*Advice to Intending Visitors to Cannes.* By H. BLANC, M.D. 1893.

From CURTIS & BEAMISH, Coventry:

*Relief.* Vol. I., No. 2. January 1st, 1893.

From DOWN BROS. London:

*Ophthalmic Atlas.* By FRANK HAYDON.

From FERET ET FILS, Bordeaux:

*Archives d'Electricité Médicale.* No. I. 15 Janvier, 1893.

From GOVERNMENT PRINTING OFFICE, Washington:
*Index-Catalogue of the Library of the Surgeon-General's Office, United States Army.* Vol. XIII. Sialagogues—Sutugin. 1892.

From CHARLES GRIFFIN & COMPANY, LIMITED, London:
*A Treatise on Peripheral Neuritis.* By JAMES ROSS, M.D. and JUDSON S. BURY, M.D. 1893.

From J. W. HATCH, Enfield:

*The Medical Pioneer.* January—March, 1893.

From H. H. HOWE, Weston, Vt., U.S.A.:

*The Abstract and Index.* November, 1892.

From THE JOHNS HOPKINS PRESS, Baltimore:
*The Johns Hopkins Hospital Reports.* Vol. III., Nos. 1, 2, 3. 1892.

From H. K. LEWIS, London:
*The British Journal of Dermatology.* January—March, 1893.
*The Middlesex Hospital. Reports of the Medical, Surgical, and Pathological Registrars for the year,* 1891. 1892.
*Hysterical or Functional Paralysis.* By. H. C. BASTIAN, M.D. 1893.
*Osteo-Arthritis.* By W. H. R. FORSBROOK, M.D. 1893.

From LONGMANS & CO., London:

*Influenza.* By JULIUS ALTHAUS, M.D. Second Edition. 1892.

From Dr. G. W. MCCASKEY, Fort Wayne:
*McCaskey's Clinical Studies.* Vol. I., No. 1. October, 1892.

From K. NADKARNI, Bombay:
*Doctors' Magazine.* December, 1892.

From OLIVER & BOYD, Edinburgh:

*The Diseases of the Fœtus.* By J. W. BALLANTYNE, M.D. Vol. I. [1892.]

From THE OPEN COURT PUBLISHING COMPANY, Chicago:

*The Open Court.* Vol. VI., Nos. 43, 46. October 27th, November 17th, 1892.

[*cont. on p.* xxiv.

From JOHN MORGAN RICHARDS, London:
*Medical Reprints.* December, 1892—February, 1893.
From G. P. ROY & CO., Calcutta:
*The Scalpel.* Vol. I., No. 3. December, 1892.
From W. B. SAUNDERS, Philadelphia:
*Notes on the Newer Remedies.* By D. CERNA, M.D. 1893.
*Medical Jurisprudence and Toxicology.* By H. C. CHAPMAN, M.D. 1892.
*A Manual of the Practice of Medicine.* By A. A. STEVENS, M.D. 1893.
From THE SCIENTIFIC PRESS (Limited), London:
*Burdett's Hospital Annual.* 1893.
From J. SHEPHERD CLARK CO., New York:
*La Revista Médico-Quirúrgica Americana.* Vol. I., Nos. 2, 4, 5. Octubre, Deciembre, 1892, Enero, 1893.
From SMITH, ELDER & CO., London:
*The More Severe Forms of Lateral Curvature of the Spine.* By NOBLE SMITH. 1892.
From W. HALE WHITE, M.D., London:
*A Method of Obtaining the Specific Heat of certain Living Warm-blooded Animals.* By W. HALE WHITE, M.D. 1892. [Reprint.]

### AMERICA.

### ARGENTINE REPUBLIC.

*Monthly.*

*Anales del Circulo Médico Argentino.* Buenos Ayres: Mariano Moreno.

### CANADA.

*Monthly.*

*The Montreal Medical Journal.* Montreal: Gazette Printing Company.

### MEXICO.

*Twice a month.*

*Gaceta Médica.* Mexico: Avenida 2 Oriente, 726.

### PERU.

*Monthly.*

*La Crónica Médica.* Lima: Carlos Prince.

### UNITED STATES.

*Weekly.*

*Medical Record.* New York: William Wood & Co.

*Medical Review.* St. Louis: J. H. Chambers & Co.

*The Cincinnati Lancet-Clinic.* Cincinnati: 199 West 7th Street.

*The Journal of the American Medical Association.* Chicago: 68 Wabash Avenue.

*The Medical and Surgical Reporter.* Philadelphia: 316-18-20 North Broad Street.

*The Medical News.* Philadelphia: Lea Brothers & Co.

*The New York Medical Journal.* New York: D. Appleton & Co.

*Fortnightly.*

*The American Practitioner & News.* Louisville: John P. Morton and Company.

*Twice a month.*

*The Medical Age.* Detroit: George S. Davis.

*Monthly.*

*American Journal of Obstetrics.* New York: William Wood & Co.

*Bulletin of the Johns Hopkins Hospital.* Baltimore: The Johns Hopkins Press.

*Index Medicus.* Detroit: George S. Davis.

*International Medical Magazine.* Philadelphia: J. P. Lippincott Company.

*Journal of Cutaneous and Genito-Urinary Diseases.* New York: D. Appleton & Co.

*The American Journal of Ophthalmology.* St. Louis: J. H. Chambers & Co.

*The American Journal of the Medical Sciences.* Philadelphia: Lea Brothers & Co.

*The Journal of Nervous and Mental Disease.* New York: 25 West 45th Street.

*The Epitome of Medicine.* New York: G. P. Putnam's Sons.

*The Post-Graduate.* New York: 226 East 20th Street.

*The Saint Louis Medical and Surgical Journal.* Saint Louis: Owens Printing Company.

*The Satellite.* Philadelphia: The F. A. Davis Company.

*The Therapeutic Gazette.* Detroit: George S. Davis.

*Quarterly.*

*The Alienist and Neurologist.* St. Louis: Ev. E. Carreras.

*Yearly.*

*Annual of the Universal Medical Sciences.* Philadelphia: The F. A. Davis Company.

*Transactions of the American Surgical Association.* Philadelphia: William J. Dornan.

*Occasionally.*

*Transactions of the New York Academy of Medicine.* New York: 12 West 31st Street.

### ASIA.

### CHINA.

*Quarterly.*

*The China Medical Missionary Journal.* Shanghai: The American Presbyterian Mission Press.

### INDIA.

*Monthly.*

*The Indian Medical Gazette.* Calcutta: Thacker, Spink & Co.

*The Indian Medico-Chirurgical Review.* Bombay: Rao & Co.

### JAPAN.

*Monthly.*

*The Sei-I-Kwai Medical Journal.* Tokio: Z. P. Maruya & Co.

### AUSTRALASIA.

### AUSTRALIA.

*Monthly.*

*The Australasian Medical Gazette.* Sydney: L. Bruck.

*The Australian Medical Journal.* Melbourne: Stillwell & Co.

### NEW ZEALAND.

*Quarterly.*

*The New Zealand Medical Journal.* Dunedin: J. Wilkie and Co.

### EUROPE

### BELGIUM.

*Monthly.*

*Bulletin de l'Académie royale de médecine de Belgique.* Brussels: F. Hayez.

### BRITISH ISLES.

*Weekly.*

*British Medical Journal.* London: 429 Strand.

*Medical Press and Circular.* London: A. A. Tindall.

*The Clinical Journal.* London: E. Knight.

*The Hospital.* London: 428 Strand.

*The Lancet.* London: 423 Strand.

*Monthly.*

*The Birmingham Medical Review.* Birmingham: Hall & English.

*The British Journal of Dermatology.* London: H. K. Lewis.

*The Journal of Laryngology and Rhinology.* London: The F. A. Davis Company.

*The Medical Chronicle.* Manchester: John Heywood.

*The Medical Magazine.* London: Southwood, Smith, & Co.

*The Practitioner.* London: Macmillan & Co.

*Edinburgh Medical Journal.* Edinburgh: Oliver and Boyd.

*The Glasgow Medical Journal.* Glasgow: Alex. Macdougall.

*The Dublin Journal of Medical Science.* Dublin: Fannin & Co.

*Quarterly.*

*Archives of Surgery.* London: J. & A. Churchill.

*The Asclepiad.* London: Longmans, Green, & Co.

*The British Gynæcological Journal.* London: John Bale & Sons.

*The International Journal of Microscopy & Natural Science.* London: Baillière, Tindall & Cox.

*The Journal of Pathology and Bacteriology.* Edinburgh: Young J. Pentland.

*The Sheffield Medical Journal.* Sheffield: Pawson & Brailsford.

*Half-yearly.*

*The Liverpool Medico-Chirurgical Journal.* Liverpool: Medical Institution.

*The Retrospect of Medicine.* London: Simpkin & Co. Limited.

*Yearly.*

*Guy's Hospital Reports.* London: J. & A. Churchill.

*St. Bartholomew's Hospital Reports.* London: Smith, Elder, & Co.

*St. Thomas's Hospital Reports.* London: J. & A. Churchill.

*The Directory of Second-hand Booksellers.* Rochdale: James Clegg.

*The Medical Annual.* Bristol: John Wright & Co.

*The Year-Book of Treatment.* London: Cassell & Company, Limited.

*Transactions of the Royal Academy of Medicine in Ireland.* Dublin: Fannin & Co.

*Year-Book of Scientific and Learned Societies.* London: C. Griffin & Co.

DENMARK.

*Weekly.*

*Hospitals-Tidende.* Copenhagen: Jacob Lund.

FRANCE.

*Three times a week.*

*Gazette des hôpitaux.* Paris: 4 Rue de l'Odéon.

*L'Union médicale.* Paris: Octave Doin.

*Weekly.*

*Bulletin de l'Académie de médecine.* Paris: G. Masson.

*Le Progrès médical.* Paris: 14 Rue des Carmes.

*Twice a month.*

*Gazette de Gynécologie.* Paris: 10 Rue Rougemont.

*Monthly.*

*Revue mensuelle de Laryngologie, d'Otologie et de Rhinologie.* Paris: Octave Doin.

*Archives cliniques de Bordeaux.* Bordeaux; Feret et Fils.

*Bi-monthly.*

*Archives de Neurologie.* Paris: 14 Rue des Carmes.

GERMANY.

*Weekly.*

*Deutsche medicinische Wochenschrift.* Berlin: Georg Thieme.

*Medicinische Bibliographie.* Leipzig: Breitkopf & Härtel.

*Monthly.*

*Fortschritte der Krankenpflege.* Berlin: H. Kornfeld.

*Quarterly.*

*Bibliotheca Medico-Chirurgica.* Gottingen: Vandenhoeck & Ruprecht.

GREECE.

*Weekly.*

**Γαληνὸς.** Athens: N. G. Passare.

HOLLAND.

*Weekly.*

*Nederlandsch Tijdschrift voor Geneeskunde.* Amsterdam: F. van Rossen.

NORWAY.

*Monthly.*

*Norsk Magazin for Lægevidenskaben.* Christiania: Th. Steens.

PORTUGAL.

*Weekly.*

*A Medicina Contemporanea.* Lisbon: José Antonio Rodrigues.

ROUMANIA.

*Twice a month.*

*Spitalul.* Bucharest: Thoma Basilescu.

RUSSIA.

*Weekly.*

*Medycyna.* Warsaw: 80 Allée de Jérusalem.

*Vrach.* St. Petersburg: C. Ricker.

*Monthly.*

*Finska Läkaresällskapets Handlingar.* Helsingfors: Helsingfors Central-Tryckeri.

SPAIN.

*Weekly.*

*El Siglo Médico.* Madrid: Magdalena, 36, 2°

*Twice a month.*

*Revista de Ciencias Médicas.* Barcelona: 9 Calle Fortuny.

SWEDEN.

*Quarterly.*

*Nordiskt Medicinskt Arkiv.* Stockholm: P. A. Norstedt & Söner.

TURKEY.

*Twice a month.*

*Gazette Médicale d'Orient.* Constantinople: A. Christidis.

